# Ethnomedicinal plants in Champadevi rural municipality, Okhaldhunga district, Nepal

**DOI:** 10.1186/s13002-023-00627-y

**Published:** 2023-12-11

**Authors:** Deepa Karki, Dipak Khadka, Ripu Mardhan Kunwar, Prakash Chandra Aryal, Hem Raj Paudel, Sijar Bhatta, Shi Shi

**Affiliations:** 1 Guangdong Key Laboratory for Innovative Development and Utilization of Forest Plant Germplasm, College of Forestry and Landscape Architecture, South China Agricultural University, Guangzhou, China; 2 GoldenGate International College, Tribhuvan University, Battispuatali, Kathmandu, Nepal; 3 Environment Protection and Study Center (ENPROSC), Baneshwor, Kathmandu, Nepal; 4 Gandaki University, Pokhara, Nepal; 5 National Herbarium and Plant Laboratories Godawari, Lalitpur, Nepal; 6 Central Department of Environmental Science, Tribhuvan University, Kirtipur, Kathmandu, Nepal

**Keywords:** Intra-cultural analysis, Medicinal plants, Traditional uses, Sociocultural transformation, Okhaldhunga, Nepal

## Abstract

**Background:**

Okhaldhunga is a hilly district with fragile socioeconomic conditions, limited access to health care, social stigma, and poor resource management, where most people rely on medicinal plants for primary health care. The use of medicinal plants for primary health care varies with socioeconomic attributes. Following the intra-cultural analysis, we documented and tested the hypothesis that use of medicinal plants in Champadevi, Okhaldhunga, Nepal, depends on socioeconomic variables.

**Methods:**

We interviewed 224 respondents, 53.12% female and 46.88% male, including 31 Brahmin, 157 Chhetri, 13 Dalit, and 23 Janajati, and conducted three focused group discussions and seven key informant interviews to record the ethnomedicinal plants used in Champadevi rural municipality, Okhaldhunga District. The relative frequency of citation (RFC) was computed to know the importance of the species. A generalized linear model (GLM) was used to see the relationship between medicinal plants reported with the sociocultural variables, which include age, gender, occupation, education, ethnicity, and religion.

**Results:**

We documented 149 medicinal plants, including 69 herbs, 22 shrubs, nine climbers, 48 trees, and one parasitic plant, belonging to 68 families and 130 genera, and used to treat 48 distinct diseases and ailments. Plant parts, leaf, and digestive disorders were frequently treated during healing. *Curcuma angustifolia* was the most cited species with RFC 0.9554. The respondents' knowledge of medicinal plant use varied significantly with age (*p* = 0.0001) and occupation (*p* = 0.003). Changes in land use, population decline of medicinal plant species, and unsustainable harvesting practices constituted the local threats to medicinal plants and associated knowledge. Elders died without passing on their knowledge to the younger generations during sociocultural transformation, and youth disinterest coupled with the free availability of allopathic medicine led to knowledge erosion.

**Conclusions:**

The use of medicinal plants in Champadevi, Okhaldhunga, was significantly depended on two socioeconomic variables age and occupation. Ethnomedicinal plants are essential in the primary healthcare system in Nepal; however, their availability and practices are declining. Thus, plans regulating land use change and human migration, acknowledging traditional healthcare practices, and raising awareness of the significance of traditional medical practices as complementary healthcare practices should be strengthened.

## Introduction

From 1515 to 2331, useful medicinal and aromatic plants have been cataloged in Nepal [1–4]. These plants are frequently valued in rural areas for food, medicine, construction, fodder, and firewood. Rural livelihood is intrigued by folklore uses for the primary healthcare system [5]. The use of ethnomedicine in rural areas has been transmitted orally from one generation to the next [6], yet it has been threatened by sociocultural transformation [7], human migration, and the limited transfer and extension of ethnomedicinal knowledge [3]. Moreover, traditional and ethnomedical knowledge are sparingly documented [8]. Geography, ethnicity, age, occupation, education, and culture substantially impact traditional ethnomedical knowledge [9]. Assessment of the interaction between the variables of geography, socio-culture, and livelihood provides ample opportunity to conserve medicinal plant species and their associated knowledge [10]. Therefore, ethnomedicinal research is required to grow and maintain medicinal plants and their associated knowledge [11, 12].

Nepal is comprised of five disparate eco-physiographical regions: the Himalayas (23% of the total area and above 5000 m asl), the High Mountains (20%, between 3000 and 5000 m asl), the Middle Mountains (30%, between 1000 and 3000 m asl), the Siwalik Hills (12.8%, between 500 and 1000 m asl), and the flat, lowlands of Tarai (13.7%, < 500 m asl) [13]. The mid-hills and mountains are home to the greatest number of medicinal plants in Nepal and are associated with diverse ethnic groups [10]. Okhaldhunga is a hilly district with fragile socioeconomic conditions, limited access to health care, social stigma, and poor resource management [14]. It is one of the understudied districts, with few ethnomedicinal publications and records of the least-used medicinal plants [10, 15].

There is no any detail investigation and documentation of medicinal in Okhaldhunga as well as in Champadevi community; therefore, this study was carried out to document and catalogue the medicinal plant used by local people of Champadevi rural municipality, to analyze the distribution of use knowledge within the socioeconomic variables such as age, gender, ethnicity, education, religion, and occupation and to assess the challenges and threats constraining the population, use, and distribution of useful plants and their associated ethnomedicinal knowledge. We hypothesized that the use of medicinal plants in Champadevi rural municipality is related to socioeconomic variables (age, gender, ethnicity, education, religion, and occupation). This information on medicinal plant species is crucial for the conservation policy and implementation. Further, to promote traditional knowledge of Champadevi community people, help to promote their identity and help in medicine development.

## Methodology

### Study area

**Fig. 1:**
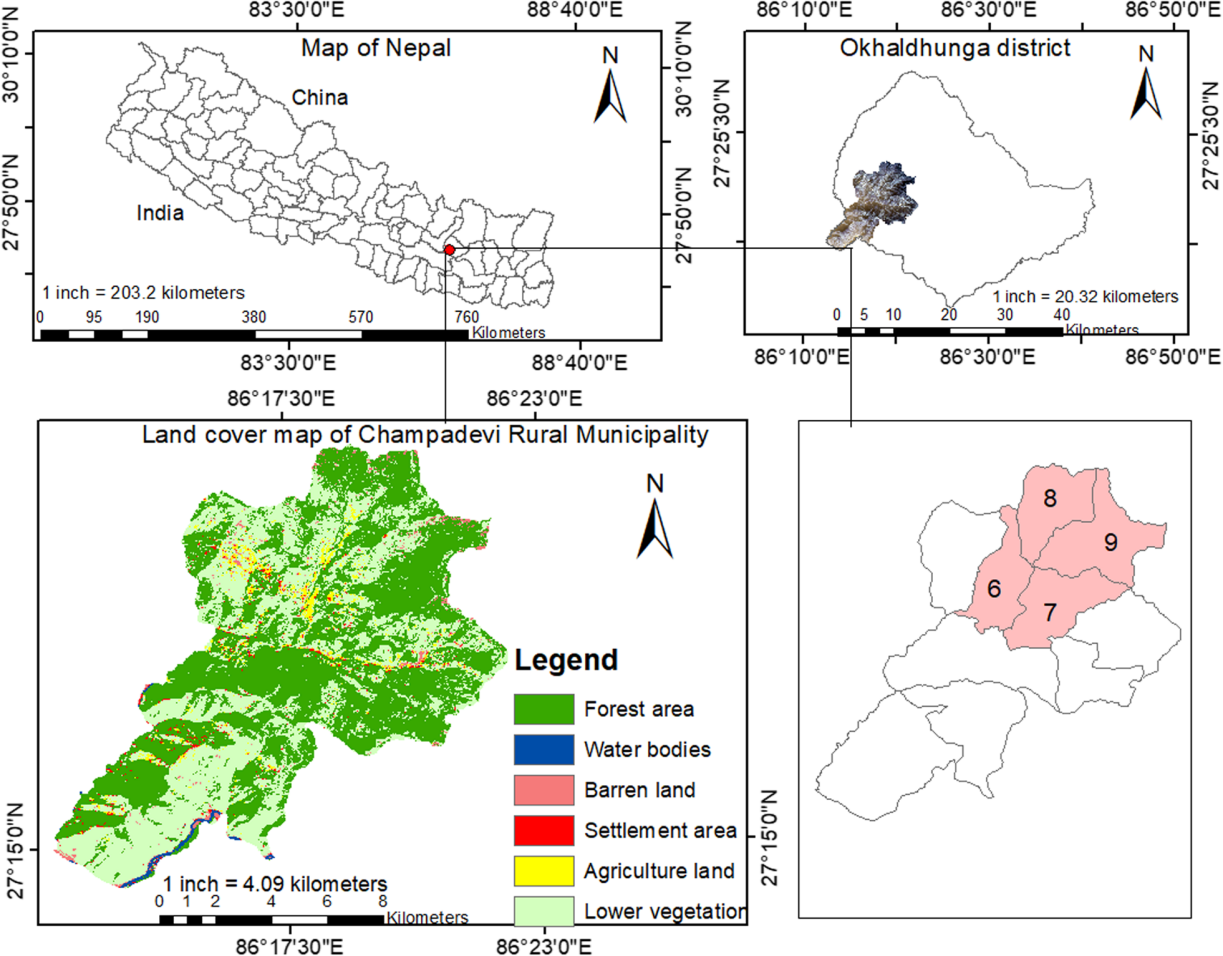
Map of the study area (Champadevi rural municipality with ward numbers 6, 7, 8, and 9)

Okhaldhunga district has 53.34% ethnic (*Janajati*) people followed by Chhetri21.22%, Brahmin 9.19%), *Dalit* (15.38%), and others (0.87%) [16]. Champadevi rural municipality is one of the southwest rural municipalities of this district bordering south to Sindhuli and west to Ramechhap district, Koshi Province, eastern Nepal. The municipality's population was 16,528 as of the 2021 Census [16]. The municipality is divided into ten wards. For this study, we selected four wards (6, 7, 8, and 9) with 1399 households where we found several traditional healers (Fig. 1).

*Bhramin*, *Chettri*, *Dalit*, and ethnic (*Janajati*) are the major ethnicity of the area. The ward numbers 6 and 7 are dominated by Chhettri, 8 has equal domination of both Chhetriand Janajati, and 9 is dominated by Janajati. People were following Hinduism and Buddhism. However, the dominated one is Hinduism. Most of the people are indigenous people of this area. The out migration rate is highest in ward numbers 8 and 9.

Champadevi village consists of mixed vegetation types. The land use of the area comprises human settlement and built-up areas (9.93%), forested areas (55.32%), barren land (5.67%), agricultural land (7.52%), water bodies (4.54%), and lower vegetation or shrubs (17.02%). Agriculture and livestock husbandry are the primary livelihood options. The primary energy source for cooking, heating, fodder, and firewood collection is nearby forests. The area is rich in traditional knowledge, and elders rural residents collect forest products and treat ailments using traditional methods. The health facility in the area is limited; people access district headquarters (Siddhicharan municipality) to treat health disorders.

## Methods

### Data collection

We adhered to the International Society of Ethnobiology's code of conduct [17]. Each respondent's verbal consent was obtained prior to the survey. The field visit occurred between July and November of 2021.

We obtained 210 or more than 210 households out of a total of 1399 households of Champadevi rural municipality ward numbers 6, 7, 8, and 9 to be surveyed following the online platform (https://www.calculator.net/sample-size-calculator.html), keeping confidence level 95% with margin of error ± 5% and household proportion 20%. Therefore, we interviewed 224 respondents from different households of Champadevi rural municipality ward numbers 6, 7, 8, and 9 following stratified random sampling methods.The socioeconomic and demographic conditions of respondents along with the use and conservation of local medicinal plants were recorded. Three focused group discussions (eight to twelve respondents in each group) and interviews with seven key informants (four Dhami/Jhakri traditional healers and three Vaidhyas) were conducted to validate the information obtained from the discussions, interviews and informal meetings. There were several informal meetings held during evenings and mornings while staying and having tea in the tea-vendor houses. The selection of key informants was based on the references made by the respondents and village secretaries. A free list of valuable medicinal plants was compiled, validated, and verified using consensus of at least three local medicinal plant experts (Dhami/Jhakri traditional healers and Vaidhyas).

### Demographic profile of the respondents

Among the 224 respondents surveyed, 105 were male (46.88%) and 119 were female (53.12%). The respondent's ages ranged from 20 to 90 years old. More than half of the respondents, 150 (66.97%), were between 20 and 59, while the remaining 74 (33.03%) were older than 60. Most respondents were Hindu (202; 90.18%) and held basic knowledge of education (137; 61.17%). Table 1 shows that most respondents were Chhetri (157; 70%), followed by Bhramin (31; 13.83%). The family's primary source of income was agriculture (176; 78.57%).

**Table 1:** Demographic profile of the respondents

Variables	Description	Total (*n* = 224)	Respondents frequency (%)
Age	20–29	24	10.71
	30–39	36	16.07
	40–49	38	16.97
	50–59	52	23.21
	60–69	42	18.76
	70–79	24	10.71
	80–89	8	3.58
Gender	Male	105	46.88
	Female	119	53.12
Education	Primary	68	30.35
	Secondary	24	10.71
	Higher secondary	17	7.59
	University	18	8.03
	Illiterate	97	43.3
Occupation	Agriculture	176	78.57
	Agri-business	16	7.14
	Services	32	14.29
Ethnicity	Bhramin	31	13.83
	Chhetri	157	70.09
	Dalit	13	5.8
	Janajati	23	10.27
Religion	Hindu	202	90.18
	Buddhist	22	9.82

### Plant collection and identification

Following Jain's [18] methodology, we gathered the voucher specimens for the final list. Each specimen was assigned the collection, place name, latitude, longitude, and code. Further consultations with plant taxonomists were conducted to verify the nomenclature of collected plant species. The identified specimens were compared to their originals at the National Herbarium and Plant Laboratories (KATH), Godawari, Lalitpur, Nepal. The voucher specimens with collection codes were submitted to the National Herbarium and Plant Laboratories (KATH), Godawari, Lalitpur, Nepal.

### Land use change analysis

The Champadevi rural municipality land use classification map was extracted from the Nepal map for land use using GIS. Landsat-7ETM of 1999, Landsat-7ETM of 2010, and Landsat-7ETM of 2020 satellite images were downloaded freely from the US Geological Survey's (USGS) Earth Explorer website (http://earthexplorer.usgs.gov). Using ArcGIS 10.4, land use change was analyzed for 1999, 2010, and 2020. Supervised classification with a maximum likelihood classifier (MLC) was used for image classification and the preparation of base maps for detecting change [19]. An accuracy assessment was done following [20].

### Quantitative and statistical analyses

Suitable quantitative methods and approaches were used in indices such as frequency of citation (FC) and relative frequency of citation (RFC) to enhance the indicative value of the ethnomedicinal study. Similar methods were used in hilly communities in India [21], whose characteristics resemble those of our study area. According to Tardio and Pardo-de-Santayana [22], the frequency of citation (FC) and relative frequency of citation (RFC) were computed.

$${\text{RFC}} = {\text{FC}}/{\text{N}}$$

where FC, number of respondents who mentioned the use of species; N, Total number of respondents who participated in a survey.

Diseases and ailments were grouped into 13 categories based on the International Classification of Primary Care (ICPC) [23]. Using a similarity index, the ethnomedicinal plants in this study and previous studies were compared [24, 25]. We compared our findings to other researchers from eastern Nepal [26–28].

$${\text{Rahman's similarity index (RSI)}} = d/\left( {a + b + c - d} \right)$$

where *a*, number of unique species in area A; *b*, number of species unique in area B; *c*, Number of common species in A and B; *d*, number of common species used for similar ailments in A and B areas.

While *a* & *b* ≠ 0 and *c* & *d* ≥ 0.

The Rahman's similarity index is used to show the cultural similarities of indigenous knowledge among the communities based on plant use knowledge [25].

Since the data of medicinal plants recorded per respondents were count data, to test the hypothesis that use of medicinal plants in Champadevi rural municipality is determined by socioeconomic variables (age, gender, ethnicity, education, religion and occupation), we used a generalized linear model (GLM) with quasi-Poisson options due to the over dispersion of the Poisson model in R 3.4.4 [29].

## Results

We documented 149 plant species representing 68 families and 130 genera. Most species belonged to the Leguminosae, Solanaceae, and Zingiberaceae families, with seven species each. Moreover, the most prevalent genus was Allium, with four species, followed by Citrus, with three. Fever (30) was treated with the most species, followed by diarrhea (28), stomachache (22), and the common cold (22). Malaria (1), eye allergy (1), nose bleeding (1), and hematuria (1) were treated the least (Table 2).

**Table 2:** Medicinal plant recorded with Family, scientific name, habit, parts, used, mode of use, FC and RFC

Family	Scientific name	Local name	Habit	Parts used	Mode of use	Ailments	FC	RFC	Voucher code
Acanthaceae	*Justicia adhatoda* L	Asuro	Shrub	Leaves	Raw, powder	Body pain, cough, asthma, tuberculosis, headache, fever, pneumonia	26	0.116	2022001
Agaricacaeae	*Agaricus campestris* L	Chate Cyau	Herb	Whole plant	cooking, eating	Blood pressure, liver disorders, diabetes	15	0.067	2022002
Amaranthaceae	*Achyranthes bidenata Blume*	Dativan	Shrub	Stem, leaves	Chewing	Tooth		0.031	2022003
Amaranthaceae	*Amaranthus viridis* L	Latte saag	Herb	Leaves	Cooking, eating	Body pain, urinary disorders, fever, asthma, liver, eye disorders	3	0.013	2022004
Amaranthaceae	*Alternanthera sessilis* (L.) DC	Bhirangi jhar	Herb	leaves	Juice, paste eating	Fever, wound	1	0.004	2022005
Amaryllidaceae	*Allium ascalonicum* L	Chyapi	Herb	Leaves	Soup drinking	Pain relief	6	0.027	2022006
Amaryllidaceae	*Allium sativum* L	Lasun	Herb	Bulb, leaves	boil with water, dried	High blood pressure, liver disorders, dysentry, intestinal worms, tuberculosis, diabetes, fever, gastric, common cold	200	0.893	2022007
Amaryllidaceae	*Allium cepa* L	Pyaj	Herb	Bulb leaves	Paste	Headache, hair growth	45	0.201	2022008
Amaryllidaceae	*Crinum amoenum* Roxb	Hare lasun	Herb	Leaves, bulb	Eating	Stomach, gastric	37	0.165	2022009
Amaryllidaceae	*Zephyranthes candida Herb*	Seto pyaj	Herb	Bulb leaves	Eating	Indigestion, gastric	4	0.018	2022010
Anacardiaceae	*Spondias pinnata* (L.f.) Kurz	Amaro	Tree	Fruit	Eating	Vomiting	1	0.004	2022011
Anacardiaceae	*Mangifera indica* L	Ampa	Tree	Leaves	Eating, paste	Stomach ache, fever	23	0.103	2,022,012
Anacardiaceae	*Choerospondias axillaris* (Roxb.) B.L. Brutt. &A.W. Hill	Lapsi	Tree	Seed	Burning seed powder	Burning wound	1	0.004	2022013
Apiaceae	*Centella asiatica* (L) Urb	Ghod tapre	Herb	Leaves	Eating	Uterus, urine infection, body pain, Upt	17	0.076	2022014
Apiaceae	*Cuminum cyminum* L	Jira	Herb	Seed	Raw	Common cold, fever, headache, stomach ache	34	0.152	2022015
Araceae	*Colocasia antiqorum Schott.var.esculenta*	Karkalo	Shrub	Whole plant	Cooking, eating	Diarrhea, body ache, iron	1	0.004	2022016
Araceae	*Acorus calamus* L	Bojho	Herb	Rhizome	Raw	Common cold, fever, headache, stomach ache	47	0.21	2022017
Asclepidaceae	*Calotropis gigantea* (L.)* Dryand*	Ank	Shrub	Stem, leaves, flower	Leaves milk eating	Diarrhea, constipation, stomach ache	1	0.004	2022018
Asclepidaceae	*Marsdenia tinctoria* R.Br	Kali lahara	Climber	leaves, flower	Eating	Pneumonia	1	0.004	2022019
Aspidiaceae	*Dryopteris cochleta* (D.Don) C. Chr	Nyuro	Herb	Leaves	Eating	Pneumonia	12	0.054	2022020
Aspidiaceae	*Dryoathyrium boryanum* (Willd.) Ching	Kalo Nyuro	Herb	Leaves	Eating	Pneumonia	1	0.004	2022021
Aspidiaceae	Athyrium filix-femina (L.) Roth ex Mert	Unyu	Herb	Leaves	Cooking, eating	Breathing problems, cough, digestive	2	0.009	2022022
Asteraceae	*Artemisia indica* Willd	Titepati	Herb	Whole plant	Juice, powder, paste	Malaria, cutting wound, headache	202	0.902	2022023
Asteraceae	*Ageratum conyzoides *L	Gandhe jhar	Herb	Leaves	Paste using	Infection, allergy	6	0.027	2022039
Barberidaceae	*Berberis aristata* DC	Chutro	Shrub	Leaves	Paste	Eye problem, jaundice	5	0.022	2022024
Basellaceae	*Basella alba* L	Lahare saag	Climber	Leaves, flower	Cooking	Body pain, kidney	1	0.004	2022025
Begoniaceae	*Begonia picta* Sm	Magar kanchi	Herb	Leaves, flower	Paste	Cutting wound, burn	2	0.009	2022026
Bombacacea	*Bombax ceiba* L	Simal	Tree	Bark	Powder paste using	Typhoid, Pneumonia	3	0.013	2022027
Brassicaceae	*Nasturtium officinale* R,Br	Sime jhar	Herb	Leaves	Eating	Immune system	1	0.004	2022028
Bromeliaceae	*Ananas comosus* (L.) Merr	Bhuikatar	Herb	Leaves, fruit	Eating	Vitamin C, immunity	4	0.018	2022029
Cannabaceae	*Cannabis sativa* L	Ganja	Herb	Leaves	Raw, powder, boiling with water	Pain relief, depression, asthma, diarrhoea	3	0.013	2022030
Cannabaceae	*Carica papaya* L	Meva	Shrub	Fruit	Fruit	Skin, cancer, good for body, increased blood	6	0.027	2022031
Caryophyllaceae	*Drymaria diandra Blume*	Abijalo	Herb	Whole plant	Eating	Jaundice, fever	3	0.013	2022032
Chenopodiaceae	*Chenopodium album* L	Bethe saag	Herb	Leaves	Cooking, eating	Digestion, body pain	14	0.063	2022033
Chenopodiaceae	*Spinacia oleracea* L	Palungo	Herb	Leaves	Cooking, eating	Stomach ache, indigestion	29	0.129	2022034
Combretaceae	*Terminalia chebula* Retz	Harro	Tree	Fruit	Boil with water, powder	Fever, tothee	39	0.174	2022035
Combretaceae	*Terminalia bellirica* (Gaertn.) Roxb	Barro	Herb	Fruit	Powder	Infection, cough, fever, liver, sore throat	35	0.156	2022036
Compositae	*Tagetes erecta* L	Sayapatri	Shrub	Flower	Powder with water	Intestinal worms, dysentery, fever, pneumonia	5	0.022	2022037
Compositae	*Guizotia abyssinica* (L.f.) Cass	Philinge	herb	Leaves, flower	Eating	Cough	1	0.004	2022038
Convolvulaceae	*Cuscuta reflexa* Roxb	Akasveli	Climber	Whole plant	Paste using juice	Jaundice, body pain, cough	21	0.094	2022040
Coriariaceae	*Coriaria nepalensis* Wall	Machine	Shrub	Leaves	Paste	Fracture	7	0.031	2022041
Cruciferae	*Brassica juncea* (L.) Czern	Rayo saag	Herb	Leaves	Cooking, eating	Body pain	15	0.067	2022042
Cruciferae	*Raphanus sativus* L	Mula	Herb	Root	Eating	Indigestion, gastric	15	0.067	2022043
Cruciferae	*Lepidium didymium L*	Chamsur	Herb	Leaves	CookingEating	Body pain	27	0.121	2022044
Cucurbitaceae	*Cucurbita Pipo* L	Pharsi	Climber	Fruit	Cooking, eating	Jaundice	16	0.071	2022045
Cucurbitaceae	*Cucumis sativus* L	Kakro	Climber	Fruit	Eating	Jaundice	9	0.04	2,022,046
Cucurbitaceae	*Momordica charantia* L	Titekarela	Climber	Fruit	Eating	Blood pressure	17	0.076	2022047
Cupressaceae	*Cupressus torulosa* D. Don	Dhupi	Shrub	Leaves	Smelling, using paste	Headache	1	0.004	2022048
Davalliaceae	*Nephrolepis cordifolia* (L)* Presl*	Pani Amala	Herb	Fruit	Eating	Cough, digestion, tonsil	2	0.009	2022049
Ericaceae	*Lyonia ovalifolia* (Wall.) Drude	Angeri pat	Tree	Leaves	Paste	Burning wounds, cutting wounds	9	0.04	2022050
Ericaceae	*Rhododendron barbatum* Wall. ex G. Don	Laligurans	Tree	Flower	Eating, powder with water	Throat problem, dysentry, diarrhea, asthma, constipation	21	0.094	2022051
Euphorbiaceae	*Phyllanthus emblica* L	Amala	Tree	Leaves, fruit	Using paste, eating	Tonsil, vitamin C	55	0.246	2022052
Euphorbiaceae	*Euphorbia hirta* L	Dudhe Jhar	Herb	Flower	Eating	Dysentery, jaundice	1	0.004	2022053
Fagaceae	*Quercus semecarpifolia Sm*	Banjh	Tree	Bark	Paste	Fracture	37	0.165	2022054
Gentiaanaceae	*Swertia chirayita* (Roxb.) H.Karst	Chiraito	Herb	Whole plant	Raw, using paste, powder, boiling with water	Fever, typhoid, blood pressure, diarrhea, dysentery, stomach ache	55	0.246	2022055
Gramineae	*Eleusine coracana* (L.)Gaertn	Kodo	Herb	Seed	Cooking with water and drinking	Bones, iron, chickenpox	5	0.022	2022056
Gramineae	*Saccharum officinarum* L	Ukhu	Shrub	Stem	Chewing	Urinary infections, jaundice	4	0.018	2022057
Gramineae	*Triticum aestivum* L	Gahu	Shrub	Leaves	Milky leaves	Blood pressure	2	0.009	2022058
Labiatae	*Ocimum basilicum* L	Tulasi	Herb	Leaves	Eating, powder with water	Coughs, dysentery, diarrhea, fever, common cold	187	0.835	2022059
Labiatae	*Mentha arvensis* L	Pudina	Herb	Whole plant	Paste, powder, boiling with water	Common cold, headache, fever	67	0.299	2022060
Labiatae	*Mentha piperita* L	Babari	Herb	Whole plant	Powder, boil powder with water	Vomiting, common cold, headache	17	0.076	2022061
Labiatae	*Perilla frutesxens* (L.) Britton	Silam	Tree	Seed	Eating	Intestinal worms, vomtiing, common cold, coughs	17	0.076	2022062
Labiatae	*Pogostemonplectranthoids* Desf	Rudilo	Herb	Whole plant	Paste	Allergy, cutting, wound	197	0.879	2022097
Lauraceae	*Lindera neesiana* (Wall. ex Nees) Kurz	Siltimur	Tree	Seed	Boiling with water and drinking	Headache, tooth pain, gastric, diarrhea, stomach ache	21	0.094	2022063
Lauraceae	*Cinnamomum zeylanicum* Breyn	Dalchini	Tree	Bark	Eating, boiling with water, and drinking	Diarrhea, gastric, depression, stomach aches, blood pressure, headache	9	0.04	2022064
Lauraceae	*Litsea monopetala* (Roxb. ex Baker) Pers	Kutmiro	Tree	bark, root	Paste	Fracture	1	0.004	2022065
Lauraceae	*Cinnammomum camphora* (L.) J.Presl	Kapur	Tree	Leaves	Eating, juice	Relief pain, common cold, eye problem	9	0.04	2022066
Lauraceae	*Persea Americana* Mill	Avocados	Tree	Fruit	Eating	Blood pressure, skin, coughs, dysentery	1	0.004	2022067
Lauraceae	*Cinnamomum tamala* (Buch.-Ham.) Ness & Eberm	Tejpat	Tree	Leaves	Paste, raw	Fever	23	0.103	2022068
Lecythidaceae	*Carea arborea* Roxb	Kyamuno	Tree	bark	Paste	Cutting wound	1	0.004	2022069
Leguminosae	*Trigonella foenum-graecum* L	Methi	Herb	Leaves	Eating	Fever, constipation, gastric body pain	30	0.134	2022070
Leguminosae	*Calopogonium mucumoides* Desv	Gahate Jhar	Herb	Leaves	Paste using	Stomach ache, bacterial infection, diarrhea	1	0.004	2022071
Leguminosae	*Bauhinia variegata* L	Koiralo	Herb	Flower	Soup drinking	Diarrhea, stomach ache	2	0.009	2022072
Leguminosae	*Butea minor* Buch.-Ham.ex Baker	Bhuletro	Shrub	Flower	Eating	Headache	1	0.004	2022073
Leguminosae	*Dolochos lablab* L	Simi	Climber	Leaves	Eating	Skin allergy	12	0.054	2022074
Leguminosae	*Acacia catechu* (L.f) Willd	Khayar	Tree	Leaves	Eating	Diarrhea, body pain	2	0.009	2022075
Leguminosae/Febaceae	*Mimosa pudica* L	Buhari Jhar	Herb	Leaves, root	Eating	Fever, urinary infections	4	0.018	2022076
Liliaceae	*Asparagus racemosus* Wall	kurilo	Herb	Stem	Eating	Pain, swelling, vitamin, milk problem in women	36	0.161	2022077
Liliaceae	*Allium hyposistum* Stearn	Jimbo	Herb	Leaves	Eating	Common cold, fever, body pain	7	0.031	2022078
Liliaceae	*Aloe vera* (L.) Burm.f	Ghuikumari	Herb	Whole plant	Leaf	Burning wound, good for skin, hair, allergy, diabetes, diarrhea	37	0.165	2022079
Liliaceae	*Paris polyphylla* Sm	Satuwa	Herb	Leaves	Eating	Snake bites	1	0.004	2022080
Loranthaceae	*Viscum articulatum* Burm.f	Hadchur	Tree	Leaves	Paste	Fracture, body pain	3	0.013	2022081
Lythraceae	*Woodfordia fruticosa* (L.) Kurz	Dhayero	Tree	Flower	Eating	Dysentery, diarrhea	7	0.031	2022082
Malvaceae	*Sida cordifolia* L	Balu jhar	Herb	Leaves	Eating	Common cold, urinary infection, intestinal worms, coughs	3	0.013	2022083
Meliaceae	*Azadirachta indica* A. Juss	Nim	Tree	Leaves, bark	Boiling with water and drinking	Intestinal worms, blood pressure, fever, common cold	43	0.192	2022084
Meliaceae	*Melia azederach* L	Bakaino	Tree	Leaves, bark	Eating	Intestinal worms	28	0.125	2022085
Menispermaceae	*Tinospora cordifolia* (Willd.)Miers	Gurjo	Climber	Stem	Dried, boiling with water	Dysentery, fever, common cold, fever, digestion, stomach ache, pressure, skin disease, diarrhea	168	0.75	2022087
Menispermaceae	*Stephania japonica* (Thunb.) Miers	Batule Paat	Climber	Leaves	Eating	Mensuration, heavy bleeding	1	0.004	2022088
Moraceae	*Artocarpus integr* (Thunb.) Merr	Rukh Kathar	Tree	Fruit	Eating	Immune system/weakness	5	0.022	2022089
Moraceae	*Ficus lacor* Buch-Ham	Kabhro	Tree	Leaves	Juice, paste eating	Gastric, fever	9	0.04	2022090
Moraceae	*Ficus religiosa* L	Pipal	Tree	Leaves	Raw	Headache	9	0.04	2022091
Musaceae	*Musa paradisiaca* L	Kera	tree	Fruit	Eating	Constipation	8	0.036	2022092
Myrtaceae	*Syzigium aromaticum* (L.) Merr. & Perry	Lwang	Tree	Flower	Raw	Immune system, asthma, pain, tooth problem	47	0.21	2022093
Myrtaceae	*Psidium guajava* L	Amba	Tree	Bark, leaves, fruit, flower	Powder boiling with water, paste	Cough, dysentery, pain relief, diabetes, diarrhoea, intestinal worm	60	0.268	2022094
Myricaceae	*Myrica esculenta* Buch.-Ham.ex D. Don	Kaphal	Tree	Fruit	Eating	Diarrhea, fever, throat disorder	7	0.031	2022095
Myristiccaeae	*Myristica fragrans* Houtt	Jayaphal	Tree	Seed, fruit	Eating	Stomach ache, indigestion, common cold, fever	21	0.094	2022096
Oleaceae	*Nyctanthes arbor-tristis* L	Parijaat	Tree	Leaves	Paste	Fever, touncil	23	0.103	2022098
Oleaceae	*Jasminum humile* L	Jai	Shrub	Leaves, flower	Juice, paste eating	Tonsil, mouth infection	35	0.156	2022099
Orchidaceae	*Dactylorhiza hatagirea* (D. Don) So	Panc aule	Herb	Tube, root	Powder, paste	Indigestion, tooth problem	15	0.067	2022100
Oxalidaceae	*Oxalis acetosella* L	Chariamilo	Herb	Leaves	Paste	Diarrhea, snake bites, fever, stomach ache	28	0.125	2022101
Pedaliaceae	*Sesamum indicum* L	Til	Herb	Seed	Raw, paste, Juice	Hair, skin, allergy	7	0.031	2022102
Phyllanthaceae	*Phyllanthus parvifolius* Buch.-Ham. ex D. Don	Paineti	Shrub	Leaves stem	Juice	Menstrual, stomach ache		0.004	2022103
Pinaceae	*Pinus roxburghii* Sarg	Khote Salla	Tree	Bark	Bark gum	Digestive, liver	3	0.013	2022104
Piperaceae	*Piper nigrum* L	Marich	Climber	Flower	Boil with water	Menstrual pain, diarrhea, depression, cancer, stomach	50	0.223	2022105
Pittosporaceae	*Pittosporum napaulense* (DC.) Rehder et Wilson	Khorsnai	Tree	Bark	Paste	Injured, pain, fracture	24	0.107	2022106
Poaceae	*Cynodon dactylon* (L.) Pers	Dubo	Herb	Leaves	Eating	Dysentery, diarrhea, headache	1	0.004	2022107
Poaceae	*Imperata cylindrica* (L.) P.Beauv	Siruphool	Herb	Leaves, flower	Juice, paste	Indigestion, diarrhoea, cutting wound	1	0.004	2022108
Polygonaceae	*Fagopyrum esculentum* Moench	Phapar	Herb	Seed, leaves	Eating	Body pain	3	0.013	2022109
Polygonaceae	*Rheum webbianum* Royle	Padamchal	Herb	Flower, leaf	Eating, using paste powder	Stomach ache, body pain, cutting, wound	4	0.018	2022110
Punicaceae	*Punica granatm* L	Anar	Tree	Fruit	Eating	Skin, unrine infection, digestive, wound	3	0.013	2022111
Rosaceae	*Prunus persica* (L.) Batsch	Aaru	Tree	Leaves, seed, fruit	Eating	Menstrual, digestion, h eal wound	4	0.018	2022112
Rosaceae	*Rubus ellipticus* Sm	Ainselu	Shrub	Leaves, root, fruit	Eating	Fever, cough, tonsil, pneumonia, chest pain	21	0.094	2022113
Rosaceae	*Rosa alba* L	Gulaph	Herb	Flower	Eating	Diarrhea, headache	5	0.022	2022114
Rosaceae	*Pyrus communis* L	Naspati	Tree	Fruit	Eating	Diabetes	1	0.004	2022115
Rosaceae	*Prunus cerasoides* D.Don	Painyu	Tree	Leaves	Eating paste	Fever, burning wound	3	0.013	2022116
Rubiaceae	*Rubia manjith* Roxb.ex fleming	Majitho	Herb	Leaves	Eating paste	Blood pressure, urine infection	7	0.031	2022117
Rubiaceae	*Adina cordifolia* (Roxb.) Brandis	karam	Tree	Leaf, bark	Eating	Stomach ache, fever, jaundice	1	0.004	2022118
Rutaceae	*Aegle mammelos* (L.) Corr	Bel	Tree	Leaves, bark, fruit, seed	Eating	Dysentery	1	0.004	2022119
Rutaceae	*Citrus x limon* (L.) Osbek	Nibuwa	Tree	Fruit	Eating	Sore throat, pressure, blood increase	15	0.067	2022120
Rutaceae	*Citrus Sinensis* (L.) Osbeck. Var. jungar	Junar	Tree	Fruit	Eating	Pain relief, headache, depression, indigestion	7	0.031	2022122
Rutaceae	*Citrus aurantifokia* (Christ).Swingle	Kagati	Shrub	Fruit	Raw, juice, boil with water	Common cold, relief, decrease fat, skin disease	46	0.205	2022123
Rutaceae	*Zanthoxylum acanthopodium* DC	Timur	Tree	Seed	Eating	Snake bites, stomach ache	25	0.112	2022124
Sapotaceae	*Madhuca longifolia* (Koenig) Mac	Mahuva	Tree	Flower	Powder, paste	Skin disease	1	0.004	2022125
Saxifragaceae	*Bergenia ciliata* (Haw.)Sternb	Pakhan bhed	Herb	Rhizome, leaf, flower	Eating	Menstrual, stomach ache, uterus	27	0.121	2022126
Solanaceae	*Capsicum frutescens* L. var. cerasiforme Bailey	Jyanmara khursani	Herb	Fruit	Eating	Gastric	34	0.152	2022127
Solanaceae	*Capsicum annuum* L	Khursani	Herb	Fruit	Eating	Indigestion, gastric	30	0.134	2022128
Solanaceae	*Datura stramonium* L	Dhaturo	Herb	Leaves	Eating	Intestinal worms, indigestion	2	0.009	2022129
Solanaceae	*Solanum surattense* Burm.f	Kanyakumari	Herb	Leaves	Eating, paste	Fever, diabetes, asthma, urine infection, tooth problem	1	0.004	2022130
Solanaceae	*Capsium frutescens* L. var. grossum Bailey	Vede Khorsani	Shrub	Fruit	Cooking	Gastric	1	0.004	2022131
Solanaceae	*Lycopersicum esculentum* Mill	Tamatar	Shrub	Fruit	Eating	Burning wound	5	0.022	2022132
Solanaceae	*Solanum erianthum* D.Don	Dhursul pati	Tree	Leaves	Eating, paste	Headache, cough	17	0.076	2022133
Theaceae	*Schima Wallichii* (DC.) Korth	Cilaune	Tree	Leaves, bark, stem	Paste	Cutting wound	3	0.013	2022134
Umbelliferae	*Daucas carota* L. var. satva DC	Gajar	Herb	Root	Eating	Intestinal worms, dysentery	2	0.009	2022135
Umbelliferae	*Carum carvi* L	Kalo jira	Herb	Seed	Raw	Fever, headache	5	0.022	2022136
Umbelliferae	*Coriandrum sativum* L	Dhaniya	Herb	Seed, leaves	Boil with water, powder	Headache, common cold	15	0.067	2022137
Umbelliferae	*Foeniculum vulgare* Mill	Madeshi Souf	Herb	Root, seed	Raw, boil with water, powder	Digestive, reproductive, body pain	57	0.254	2022138
Umbelliferae	*Trachyspermum ammi* (L.) Sprague	Jvano	Herb	Seed	Dry powder, boil with water	Diarrhea, stomach ache, common cold, fever, pain, immunity	56	0.25	2022139
Urticaceae	*Urtica dioica* L	Signup	Shrub	Leaves	Eating	High pressure, body pain, gastric, vitamin A jaundice	40	0.179	2022140
Valerianaceae	*Valeriana jatamansii* Jones	Sughandawal	Herb	Whole plant	Smelling, using paste	Depression, Headache	1	0.004	2022141
Verbenaceae	*Premna integrifolia* L	Gidari	Shrub	Bark, root, stem, leaves	Juice	Typhoid, stomach ache	12	0.054	2022142
Verbenaceae	*Callicarpa macrophylla* Vahl	Guyallo	Shrub	Eating	Fruit, flower	Pneumonia	5	0.022	2022143
Zingiberaceae	*Zingiber officinale* Rosc	Aduwa	Herb	Rhizome	Powder, pate, juice, boil with water	Diarrhea, dysentry, nausea, tonsil, common cold, gastric	203	0.906	2022144
Zingiberaceae	*Curcuma angustifolia* Rpxb	Besar/Haledo	Herb	Rhizome	Raw, boiling with water, powder with water	Diarrhea, dysentery, cutting wound, allergy, tonsil, fever, common cold, digestion	214	0.955	2022145
Zingiberaceae	*Kaempferia* rotunda L	Bhuin champa	Herb	Flower	Eating	Cancer, Swellings, Cuts,	1	0.004	2022146
Zingiberaceae	*Costus speciosus* (Koenig.) Sm	Betlauri	Shrub	Flower, bud, leaf	Eating	Fever, intestinal worm, urine infection	2	0.009	2022147
Zingiberaceae	*Amomum subulatum* Roxb	Alainchi	Tree	Fruit	Boil with water, milk, powder	Diarrhea, vomiting cough	45	0.201	2022148
Zingiberaceae	*Curcuma caesia* Roxb	Kalohaledo	Herb	Rhizome	Paste, boil with water, powder	Pneumonia, stomach ache, common cold, fever, headache	25	0.112	2022149
Zingiberaceae	*Elettaria cardamomum* Maton	Sukhmel	Tree	Fruit	Eating	Oral disease, asthma, diarrhea, kidney disorder	34	0.152	2022150

### Habit with the plant parts used in medicine

**Fig. 2:**
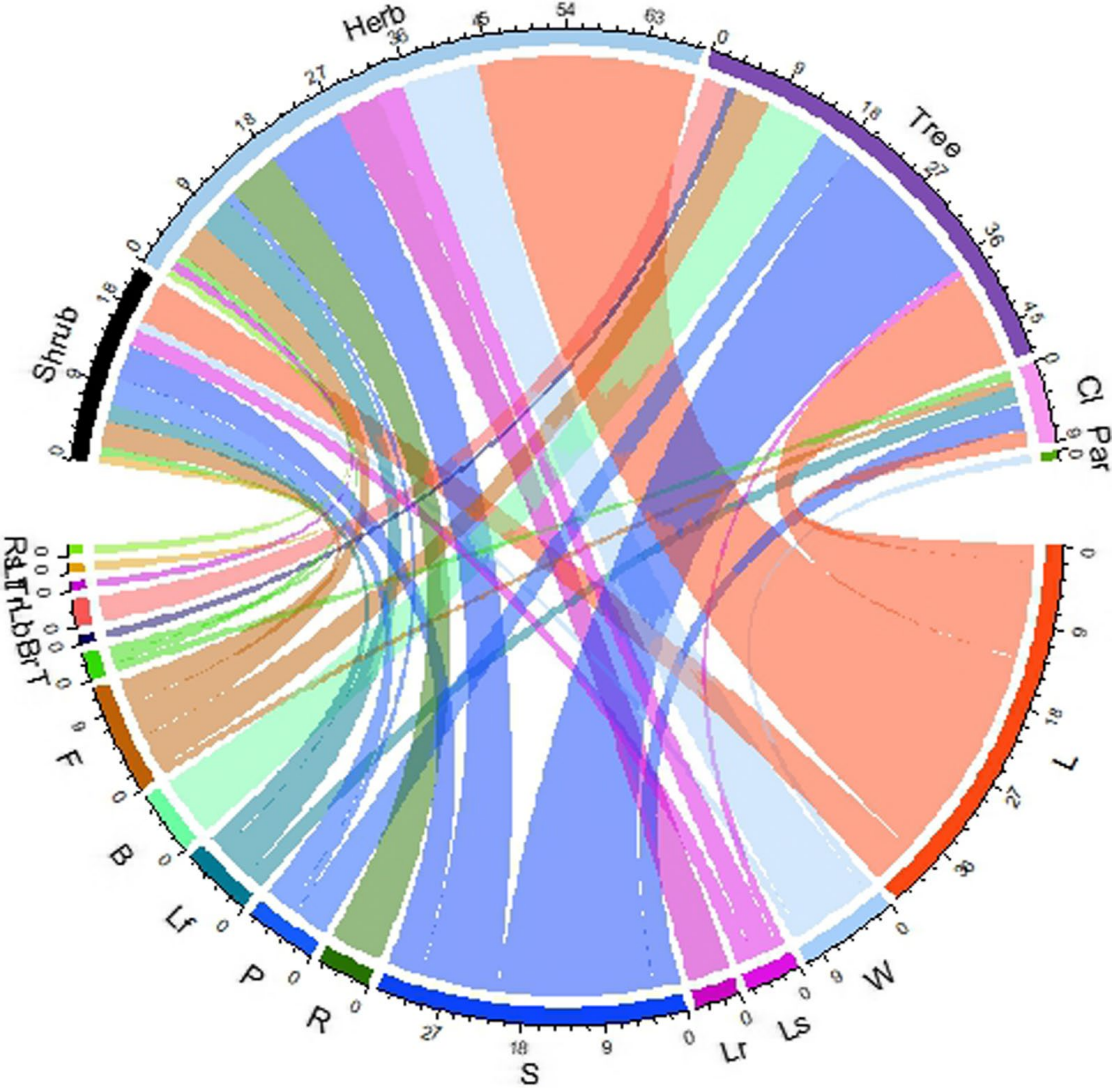
Habit-wise parts of the plant used to treat diseases (where Rs = root/rhizome/bulb, seed/fruit, Lt = leaves, trunk, Lb = Leaves, bark, Br = bark, root/rhizome/bulb, T = stem, F = flower, B = bark, Lf = leaves and flower, P = bark, root, stem, leaves/leaves, bark, stem/root, leaves, flower/leaves, root, stem/bark, leaves, stem, fruits/stem, leaves, fruits, R = root/rhizome/bulb, S = fruits/seed, Lr = leaves, root, Ls = leaves, seed/fruit, W = whole plants, L = leaves, Par = parasites, Cli = climber)

Most recorded species, 69 (46.3%), were herbs, followed by trees, 48 (32.21%), shrubs, 22 (14.77%), climbers, 9 (6.04%), and parasites, 1 (0.67%). Nearly all plant parts were used in ethnomedicine. However, the most frequently used plant parts were leaves in 74 (49.67%) of the species, followed by fruit 30 (20.13%), flower 19 (12.75%), seed 11 (7.38%), whole plant 11 (7.38%), bark 11 (7.38%), root 7 (4.69%), stem 6 (4.02%), rhizome 5 (3.35%), and bulb 4 (2.68%) (Fig. 2).

### Relative frequency of citation (RFC)

**Fig. 3:**
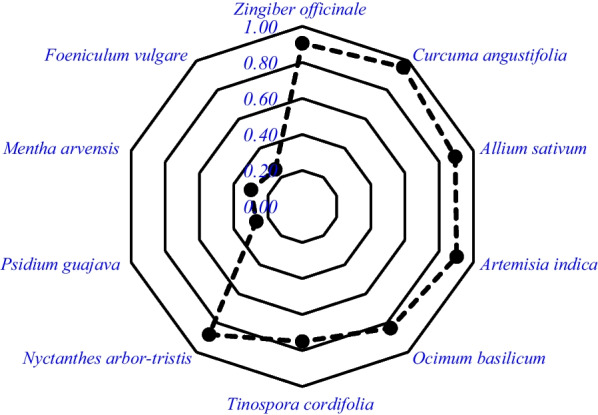
Top ten ranked plant species reported by respondent where there are ten radar lines one radar represent 0.1 and goes up to 1. The black solid point is the RFC value of the species

The relative frequency of medicinal plant citations ranged from 0.004 to 0.955, with *Curcuma angustifolia* (0.9554) having the highest frequency, followed by *Zingiber officinale*. (0.9063), *Artemesia indica* (0.902), *Allium sativum* (0.893) and *Nyctanthes arbor-tristis* (0.879) (Fig. 3).

### List of plants with diseases treated

The recorded 149 medicinal plants were used to treat 48 disorders and 13 disease categories. The greatest number of medicinal plants (119) was employed to treat digestive disorders (Table 3).

**Table 3:** List of plant species used for specific categories of disease and ailments

Ailments categories	Local terms/emic use reports (biomedical term)	Number of species
Circulatory	Rakta chap badeko (blood pressure)	17
	Mirgaula ramro banauxa (help Kidney function)	
	Kalejo ramro banauxa (help liver function)	
Digestive	Kabjiyet/ disa garna garo huda (constipation)	119
	Pakhala/Cherpati Lageko (diarrhea)	
	Aaun parda (dysentery)	
	Aapach/Khana naruchda (indigestion)	
	Pet ma Juka parda (intestinal worms)	
	Pahelo rog (jaundice)	
	Pet dukhda (stomachache)	
	Banta/ulti huda (nausea, vomiting)	
	Amilo pani aune/Chati dukhda (Gastritis)	
Endocrine	Chini rog/Sugar (diabetes)	7
Eye	Aankha ka samasyaharu huda (eye complaints)	3
	Aankha pakda (eye allergy)	
General and unspecified	Jooro (fever)	37
	Machet/ kira le tokda (malaria)	
	Jwaro, banta, tauko dukhda, diarrhea (typhoid)	
	Sarpa le tokda (snakebite)	
Genetic disorder	Gatha, Girkho, chala palauda (cancer)	3
Mental illness	Tanab/Dhapedi huda/ Jharko lagda (depression)	5
Musculoskeletal	Jiu dukheko (bodyache)	46
	Haddi kamjori (bone weakness)	
	Haddi vachiyeko/Futeko (fracture)	
	Kamjor huda (weakness)	
	Haad/Jorni dukhda (arthritis)	
	Jyan Dukhda kheri (body pain)	
	Dant/Gija Dukhda (toothache)	
Neurological	Tauko dukhda (headache)	15
Post-partum hemorrhage	Dherai ragat bagda (menstrual bleeding)	7
	Pathe ghar dukhda (uteral disorder)	
Respiratory	Dam/Sas ferna garo huda (asthma)	66
	Ruga/Khoki (common cold)	
	Khasi (cough cold)	
	Naak bata ragat bagda (nose bleeding)	
	Fokso ko samsya huda (pneumonia)	
	Fokso/chati dukhda (tuberculosis)	
	Ghanti Basda/dhukda (sore throat, tonsillitis)	
Skin	Luto auda (scabies)	45
	Poleko/dadeko (burn, scalds)	
	Kateko (cuts and injuries)	
	Kapal jharda (hair fall)	
	Sarir ma daag auda (skin infection)	
	Chala rog (skin fungal diseases)	
Urinary system	Pisab Polda/ragat dekhida (Hematuria, uric acid problem)	10
	Pisab pahelo (urine infections)	

### The use of medicinal plants varies with the sociocultural variables

**Fig. 4:**
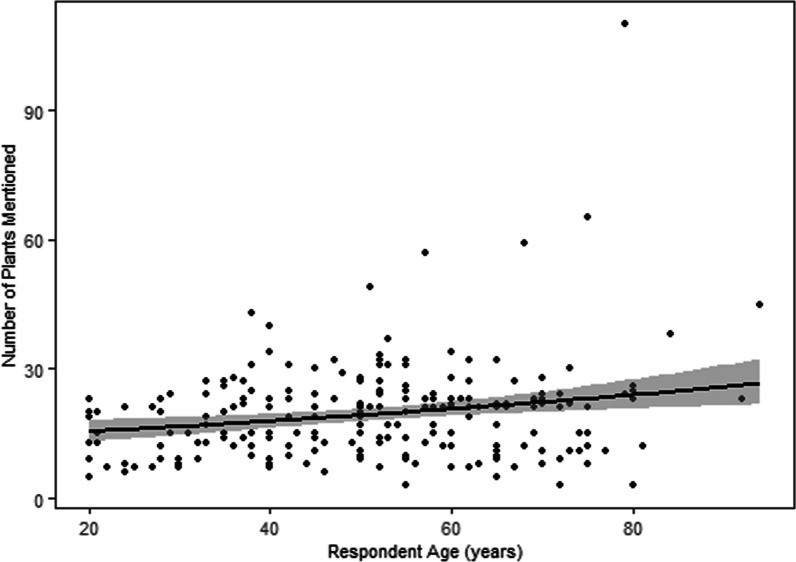
Number of plants reported with the age of the respondents

Age and occupation were the only variables among age, gender, education, occupation, ethnicity, and religion significantly associated with the number of plants reported (Table 4). Figure 4 demonstrates that older individuals mentioned more medicinal plants (*p* = 0.0001) than younger individuals. People in the agri-business sector reported significantly (*p* = 0.003) more medicinal plants than those in other occupations.

**Table 4:** Summary table of the generalized linear model (GLM) with quasi-Poisson showing knowledge about the number of medicinal plants among the respondents where sign * indicates significance

Variables	Estimate	Std.Error	t-value	p-value	Lo.CI	Up.CI
Intercept	8.716	2.714	3.212	0.002	3.397	14.035
Age (years)	0.191	0.048	3.956	0.0001*	0.096	0.286
Occupation_agri-business	8.607	2.908	2.959	0.003*	2.906	14.308
Occupation_Service	2.956	2.174	1.36	0.175	− 1.305	7.218

### Status of medicinal plants and their traditional knowledge

The major challenges and threats in ethnomedicine were the migration of young people to urban areas, land use change in the village, and a decreasing use of forest products. Respondents reported a decrease in the population of some plants (21) as a threat to conservation caused by untrained and unprofessional collection. The sharing of ethnomedicinal knowledge was believed to decrease the effectiveness of medicine; therefore, all the traditional healers (Dhami and Jhakri) and three Vaidhya (Ayurvedic physicians) were not effortlessly passing their knowledge to the younger generation. Further, the hegemony of allopathic medicine is cited as a concern for limiting ethnomedicine. From observation, it was found that in the entire surveyed area, only one botanical to distribute medicinal plants and products; however, there were four health centers to sell allopathic medicine. Similarly, youth were found to have a diminished interest in ethnomedicine. For instance, we recorded an average of 20 species per respondent, with 20- to 29-year-olds reporting an average of 14 plant species.

Changes in land use patterns also represent a significant obstacle for using ethnomedicinal plants. Two (1.34%) of the 149 species recorded were purchased from a market, while the remaining 147 (98.65%) were collected from agricultural land, 76 (51%), forest, 49 (32.89%) and fallow/transition land, 22 (14.77%). The land use change analysis revealed a 56.67% decrease in shrubland area and a 32.05% decrease in agricultural land area (Table 5), limiting the availability of medicinal plants. Change in land use also prevailed the dissented medicinal plants picking sites and traditional harvesting calendars.

**Table 5:** Summary of LULC change in the period 1999–2020 in hectares

LULC/Year	1999	2010	2020	Overall change in 21 years
Forest area	45 (35.46%)	57.6 (45.39%)	70.2 (55.32%)	+ 56% (1.2 ha^−1^)
Water bodies	8.1 (6.38%)	4.86 (3.83%)	5.76 (4.54%)	− 28.88% (0.11 ha^−1^)
Barren land	5.4 (4.26%)	4.95 (3.90%)	7.2 (5.67%)	+ 3.33% (0.08 ha^−1^)
Settlement area	4.5 (3.55%)	8.1 (6.38%)	12.6 (9.93%)	+ 180% (0.38 ha^−1^))
Agriculture land	14.04 (11.06%)	15.39 (12.13%)	9.54 (7.52%)	− 32.05% (0.21 ha^−1^)
Shrub land	49.86 (39.29%)	36 (28.37%)	21.6 (17.02%)	− 56.67% (1.34 ha^−1^)

## Discussion

We recorded more ethnomedicinal plants than previous studies from eastern Nepal [27, 30–32]. In indigenous community-centered studies, [30] focused on the Lepcha community of Illam district, eastern Nepal, and recorded 35 species, while the Rai community-focused study in the Bhojpur district, eastern Nepal, recorded 35 species [32]. A study from Kavrepalanchok District, central Nepal, recorded 116 species [33]. A study from the Machhapuchchhre rural municipality of Kaski District, Nepal, recorded 105 species [23]. A study from far west Nepal recorded 135 species [34]. A study conducted in villages of central and western Nepal reported 192 medicinal plants [35]. This indicates that the Champadevi area is rich in medicinal plant knowledge, similar to other parts of Nepal, which might be related to the geographical uniqueness and remote area inhabiting indigenous people lacking medical resources [36].

Our research demonstrated a similarity index of 0.077 to Bhattarai and Khadka [26], 0.054 to Bhattarai [27], and 0.050 to Shrestha et al. [28]. Least similarity indices (0.07–0.05) indicate that more unique species have been recorded in Okhaldhunga district, revealing that people rely more on locally available medicinal plants to treat illness.

The differences in the use of species may be attributable to the people's socioeconomic status, including a link to national roads and health facilities, as well as awareness [23] and easy access. The differences in the use of species may be bonded to the people's socioeconomic status, including a link to national roads and health facilities, as well as awareness. It is evident that the people of remote and hilly, Nepal, have extensive knowledge of medicinal plants [10].

### Plant parts used and their growth forms

Among the choices of plant parts, leaves, fruits, and flowers were most frequently collected and utilized. Due to their more frequent collection than roots and bark, leaves were also harvested and processed to create various mixtures. The leaves of a plant are the most sensitive because they contain the highest concentrations of bioactive secondary metabolites and play an essential role in the plant's defense system [37]. Additionally, the preparation of leaf extracts preserves the drug's active components more effectively than other plant parts [38]. In contrary, root contains more bio active compounds [39].

Herbs constituted the majority of collected and utilized species (46%), followed by trees (32.67%). As tree leaves and herbs were frequently valued and apparent trees and abundant herbs were primarily selected, ecological traits were followed in the selection of medicinal plants. However, the random selection was prevalent on other parts of the country [40, 41]. Irrespective to our hypothesis, plant collection was influenced more by the ecological traits (abundance and apparency). As herbs are simple to cultivate and abundant, they are easy to harvest, process, and prepare for pharmacological consumption [42]. It is believed that the medicinal benefits of a plant increase with its abundance [43]. Moreover, obvious or salient plants are frequently collected [8]. In addition, secondary metabolites are more abundant in herbs [44].

### Medicinal plants use and sociocultural variables

Our hypothesis tested yielded significant relationship with the age and occupation group among the socioeconomic variables age, gender, education, occupation, ethnicity, and religion. Older respondents reported more plants than younger ones. It may be due to the elders' increased plant knowledge. Nonetheless, this may be more than a mere factor, as plant knowledge is linked to social context [45]. Older generations serve as custodians of traditional knowledge, are more familiar with traditional treatments, and have limited exposure to modern medical procedures [46]. It may also be attributable to the younger generation's disinterest in traditional medicine and related to the time they spend with their elders. Young people are highly mobile in pursuing opportunities [47]. Agri-businessmen reported more medicinal plants than other respondents (service men). These businessmen are locally engaged in the medicinal plant industry, local medicinal plant expert products, and food-related businesses, which may have aided them in acquiring a deeper understanding of medicinal plants. It is evident that people engaged in the medicinal plant business have more medicinal knowledge [48]. As medicinal plant use is more dependent on family background and the transfer of plant knowledge is dependent on family, business people may have had an excellent opportunity to converse about plants at home [49].

### Conservation of medicinal plants and their traditional knowledge

Due to rapid population growth, poverty, a lack of valuation of ecological services, and ignorance of biophysical limitations, the area of lower vegetation (herbs and shrubs) and anthropogenic landscape have decreased due to human activities, including settlement and built-up areas. This change has altered the region's physical landscapes and ecosystem services [50]. Population of 21 species (*Drymaria diandra, Curcuma caesia*, *Basella alba*, *Achyranthes bidenata*, *Bombax ceiba, Cuscuta reflexa*, *Bergenia ciliata*, *Carea arborea*, *Swertia chirayita*, *Butea minor*, *Viscum articulatum*, *Woodfordia fruticosa*, *Adina cordifolia, Premna integrifolia, Rheum webbianum*, *Mangifera indica*, *Terminalia chebula*, *Terminalia bellirica*, *Callicarpa macrophylla*, *Melia azederach*, and *Marsdenia tinctoria* were reported to have declined due to land use change followed by a change in agricultural area. Evidently, land use change alters plant use patterns and promotes more use of resources from the secondary forest and the use of non-indigenous species [7].

For ethnomedicine, the sociocultural transformation may be one of the significant threats and challenges [51], which includes the migration of youth to urban areas in search of good opportunities [32] and the disinterest of young people in traditional medical practices, similar to other global records [52]. Typically, older people are the source of ethnomedicine, but sometimes, they pass away without passing on their knowledge to the younger generation, which poses a significant threat to ethnomedicine [53]. A study from Nepal revealed that youths are less interested in ethnomedicine [21], which is another threat to ethnomedicine. However, rural people prefer to retain their knowledge of medicinal plants [54]. There is a belief that sharing ethnomedicinal plant use knowledge diminishes healing effectiveness, so most local healers in Nepal wish to keep their ethnomedicinal knowledge secret. However, most of them impart their knowledge to close relatives, such as sons, daughters, and daughters-in-law.

The dominance of allopathic medicine is also a challenge for ethnomedicine, given that allopathic medicine is readily available and believed to have a rapid healing capacity [55]. Traditional healers should impart traditional knowledge to the younger generation to preserve this knowledge. The valuable knowledge of ethnomedicine should be preserved, and young people should be made aware of the ethnomedicinal system. A mechanism for intergenerational learning should be established [56] by organizing interaction programs for younger and older villagers and households.

## Conclusions

We recorded 149 medicinal plants from 68 families and 130 genera used to treat 48 diseases. The most plants were used for digestion (119), while the fewest were used for genetic disorders (3). *C. angustifolia* was the most frequently cited medicinal species, followed by *Z. officinale*, *A. sativum, A. indica,* and *O. basilicum*. Our study supported the positive relationship of medicinal plant use knowledge with the elder people and people involve in agri-business. Changes in land use, population decline, and unsustainable medicinal plant harvesting practices posed the greatest threats to medicinal plant conservation in Okhaldhunga. The major threats to medicinal plants and their knowledge are sociocultural transformation, vertical transfer of plant knowledge, and youth disinterest. We suggest managing ongoing land use change and human migration and educating individuals on the traditional medical system.

## Data Availability

All the data used in this study are used in the manuscript.

## Abbreviations

CBS: Central Bureau of Statistics
FC: Frequency of citation
FGDs: Focus group discussions
GIS: Geographic Information System
GLM: Generalized linear model
ICPC: International classification of primary care
KATH: National Herbarium and Plant Laboratories
LRMP: Land and resource management
MLC: Maximum likelihood classifier
RFC: Relative frequency of citation
RSI: Rahman's similarity Index
USGS: US Geological Survey's
